# Adaptive Intrawell Matched Stochastic Resonance with a Potential Constraint Aided Line Enhancer for Passive Sonars

**DOI:** 10.3390/s20113269

**Published:** 2020-06-08

**Authors:** Haitao Dong, Ke He, Xiaohong Shen, Shilei Ma, Haiyan Wang, Changcheng Qiao

**Affiliations:** 1 School of Marine Science and Technology, Northwestern Polytechnical University, Xi’an 710072, China; haitaodong@mail.nwpu.edu.cn (H.D.); hk@nwpu.edu.cn (K.H.); xhshen@nwpu.edu.cn (X.S.); slma@mail.nwpu.edu.cn (S.M.); 2 Key Laboratory of Ocean Acoustics and Sensing (Northwestern Polytechnical University), Ministry of Industry and Information Technology, Xi’an 710072, China; 3 School of Electronic Information and Artificial Intelligence, Shaanxi University of Science and Technology, Xi’an 710021, China; 4 The 27th Research Institude of China Electronic Technology Group Corporation, Zhengzhou 450047, China; ccqiao1227@sina.com; 5 Zhengzhou Key Laboratory of Underwater Information System Technology, Zhengzhou 450000, China

**Keywords:** adaptive stochastic resonance (ASR), matched intrawell response, nonlinear filter, line enhancer, autonomous underwater vehicles (AUVs)

## Abstract

Remote passive sonar detection and classification are challenging problems that require the user to extract signatures under low signal-to-noise (SNR) ratio conditions. Adaptive line enhancers (ALEs) have been widely utilized in passive sonars for enhancing narrowband discrete components, but the performance is limited. In this paper, we propose an adaptive intrawell matched stochastic resonance (AIMSR) method, aiming to break through the limitation of the conventional ALE by nonlinear filtering effects. To make it practically applicable, we addressed two problems: (1) the parameterized implementation of stochastic resonance (SR) under the low sampling rate condition and (2) the feasibility of realization in an embedded system with low computational complexity. For the first problem, the framework of intrawell matched stochastic resonance with potential constraint is implemented with three distinct merits: (a) it can ease the insufficient time-scale matching constraint so as to weaken the uncertain affect on potential parameter tuning; (b) the inaccurate noise intensity estimation can be eased; (c) it can release the limitation on system response which allows a higher input frequency in breaking through the large sampling rate limitation. For the second problem, we assumed a particular case to ease the potential parameter $a_{opt}=1$. As a result, the computation complexity is greatly reduced, and the extremely large parameter limitation is relaxed simultaneously. Simulation analyses are conducted with a discrete line signature and harmonic related line signature that reflect the superior filtering performance with limited sampling rate conditions; without loss of generality of detection, we considered two circumstances corresponding to $H_{1}$ (periodic signal with noise) and $H_{0}$ (pure noise) hypotheses, respectively, which indicates the detection performance fairly well. Application verification was experimentally conducted in a reservoir with an autonomous underwater vehicle (AUV) to validate the feasibility and efficiency of the proposed method. The results indicate that the proposed method surpasses the conventional ALE method in lower frequency contexts, where there is about 10 dB improvement for the fundamental frequency in the sense of power spectrum density (PSD).

## 1. Introduction

Passive sonars have been proven to have practical efficiency in detecting and recognising selfemitting underwater targets such as ships, submarines, and autonomous underwater vehicle (AUVs), etc. [1,2,3]. Generally, narrowband discrete components of ship-radiated noise, known simply as lines or tonals, have largely prevailed so far [4,5,6]. From the view of rotatory machinery, ship-radiated line signatures that are relevant to the engine and shaft/propeller rotation attract lots of attention, of which the long-term challenging problem is to address the weak signatures from heavy noise background in the far field scenario [3,7,8]. In this way, increasing the signal-to-noise ratio (SNR) is expected as a tenet to detection performance for applications. To enhance the narrowband discrete components, passive sonars usually employ an adaptive line enhancer (ALE) as a pre-processing step [9,10,11,12]. However, performance of the conventional ALE is limited, and advanced signal processing techniques dedicated to better denoising performance are of highly practical importance.

In general, more noise in the system often leads to worse detection performance and degrades the estimation accuracy. However, despite its disruptive character in nature, noise does play an important constructive role under certain conditions [13]. This phenomenon so called stochastic resonance (SR), in which the output periodic signals can be enhanced by the cooperative effect between “signal”, “noise” and certain “nonlinear systems” [14]. Since proposed by Benzi et al. in 1981 [15], it has become a hot research topic in the field of nonlinear science. Previous studies have addressed a lot of research progress, both theoretical and experimental, in achieving some noteworthy contributions in practical applications [8,16,17,18,19,20,21]. As a result, taking SR for weak signal detection is regarded as a potential novel technique for weak signal detection, especially under low SNR conditions.

Classical SR theories generally refer to a noise enhanced phenomenon by means of adding an appropriate amount of noise [14]. This is restricted to applications of how to remove proper noise, especially under low SNR circumstances. Rather than adjusting the input noise levels, Xu et al. [22] proposed a parameter-induced stochastic resonance (PSR) which highlighted the effect of the nonlinear systems and promoted the flexibility of SR utilization in designing systems to deal with the noise. In view of this, utilizing SR in weak signal processing could be considered as a special nonlinear filter, which can achieve superior performance compared with the traditional noise-suppression-based filters [8,23,24,25]. Among the publications on this point, the simplest first-order overdamped Langevin equation (LE) model is largely adopted, while the filtering performance is limited [8,26,27]. To get a cleaner filtered signal with higher signal-to-noise ratio (SNR), the second order Duffing equation with an underdamped system is used for a secondary filtering effect, which reflects a superior filtering performance compared with the traditional state-of-the-art methods [23,24,28]. For the purpose of better improvements, some authors reported cascaded SR systems [29,30], coupled SR system [31,32], etc. with bistable or multi-stable potentials. As a matter of fact, superior performance generally required a high sampling frequency condition to fully take advantage of the nonlinear property by concentrating most of the noise energy into the low-frequency region [33]. Several efforts have addressed the problem of large parameter stochastic resonance (LPSR) by tuning the signal structure and (or) the system parameters [24,27,34]. However, all the methods of LPSR are based on the classical SR part, and the classical SR generally requires a large sampling frequency that is more than 50 times the driving frequency. All these processing approaches are essential to deal with the periodic signal individually, and hence, flexible limited and lack of computation efficiency. An open problem for practical applications is “Can the SR be realized under limited sampling rate?”

To address this problem, limited studies on intrawell SR can give us inspirations. Actually, intrawell SR can exist for a periodically driven noisy signal in terms of underdamped bistable nonlinear dynamic systems [14]. Alfonsi et al. [35] gave a phemonological nonadiabatic description of intrawell SR by adding Lorentz’ colored noise. On the basis of this, Li et al. [36] further demonstrate that the intrawell SR phenomenon usually occurs in a system with optimal system parameters. Since the system response speed of intrawell motion is much larger than that of interwell, it can release the limitation on system response speed which allows a higher input frequency. In this way, the large sampling condition can be eased in properly controlling the escape time scale (characterizing interwell jumps) together with the relaxation time of the particle. Back to the view of potential parameter tuning, the adjustable potential parameters can be equivalent to tuning potential depth and well separation [28]. There is lack of flexibility in matching the escape time scale and the relaxation time only with the damping effect. In our previous work [37], the nonlinear filtering effects of intrawell matched stochastic resonance is analyzed, which have shown a superior filtering performance as well as a wider range of frequency response. This can give us a guidance to ease the large sampling frequency limitation.

On the basis of the aforementioned analyses, this paper proposes a novel parameterized filter implementation on adaptive intrawell matched stochastic resonance (AIMSR) with a potential constrained Duffing system. As can be seen in [24,37] matching a high frequency signal requires extremely large system parameters. This refers to a large range of parameter searching interval to limit the computational efficiency and parameterized realization, especially in an embedded system. In this regard, we further eased the potential parameter a=1 which makes it practically realizable. The nonlinear filtering effects are analyzed and evaluated, reflecting a superior performance in enhancing the lines especially under a limited sampling rate. Application verification is further conducted with a set of low sampled data fragment of autonomous underwater vehicles (AUVs). The output performance is further compared with the conventional ALE method, which shows an excellent filtering performance in enhancing the lines, especially for the lower frequency fundamental signature.

The rest of the paper is arranged as follows. After introducing the signal model and measurement in Section 2, in Section 3, the theory and implementation of the AIMSR method are detailed and described with a potential constraint Duffing system. Section 4 verifies the validity of utilizing AIMSR in dealing with the single and muli-harmonic line signature signals. Section 5 verifies the practicability of the proposed method by analyzing a set of low sampled AUVs data fragments. Besides, intensive discussions are made to give an insight into the principal results and inspired future investigations. Finally, concluding remarks are drawn in Section 6.

## 2. Signal Model and Measurement

Ship radiated noise have been studied for years. Ross and Urick have given an excellent description of the mechanisms of sound generation by large surface ships and submarines [4,5]. They have shown that the radiated noise from a ship is a combination of broadband noise and sinusoidal tonal signals that are generated by many sources. Theoreitical and experimental results have shown signatures related to the rotation of engines, shaft-line dynamics, propeller cavitations are mostly efficient for the purpose of passive detection, tracking, and classification [1,6,38,39]. From the view of rotatory machinery of the shaft and propeller, the harmonic signature is typically connected to the running state and can be measured by vibrational and acoustic approaches. For an underwater surveillance system, to address the shaft and propeller signatures, algorithms such as the Detection of Envelope Modulation on Noise (DEMON) [40], cyclic modulation coherence (CMC) [7], etc. are employed to check for the presence of periodic components to the broadband cavitation noise. This generally requires acoustic measurement of broadband noise in a high-frequency range of thousands of Hertz. Additional studies in recent years have shown that the harmonic signature as a propeller rotates can be observed by low frequency acoustic/vibrational measurement as well, which may propagate for a very long distance [41]. Such measurement techniques have been ubiquitously seen in sonobuoys, ocean bottom seismometers (OBS), acoustic vector hydrophone and other small-scale equipment [41,42,43].

As a matter of fact, these sinusoidal tonals are commonly considered to be the “acoustic fingerprint” of a moving vessel. Table 1 shows the major contributions and relations to the sinusoidal tonal signals from ship engine and propeller. In the situation of practical applications, these sinusoidal components can be modeled as follows,

(i) Discrete line signature

(1) $$\begin{array}{cc} r(t) & =s(t)+n(t)\\ \; & =A_{0}cos\left(2\pi f_{0}t+\varphi_{0}\right)+n\left(t\right) \end{array}$$

where $f_{0}$ represent the character frequency, $A_{0}$ and $\varphi_{0}$ are the corresponding amplitude and phase, respectively. n(t) represent the combination of radiated broadband noise and ambient noise.

(ii) Harmonic related line signature

(2) $$\begin{array}{cc} r(t) & =s(t)+n(t)\\ \; & =\sum_{h=1}^{M}A_{h}cos\left(2\pi hf_{0}t+\varphi_{i}\right)+n\left(t\right) \end{array}$$

in which *h* is the harmonic number, $A_{h}$ and $\varphi_{h}$ are the amplitude and phase of the *h*th harmonic component, and $f_{0}$ is the fundamental frequency of shaft rotation, n(t) represent the combination of radiated broadband noise and ambient noise.

For small targets such as AUVs, speedboats, etc. with rotation marine engine and propeller, the signatures are in a periodic pattern as well. Their acoustic characteristics have been comprehensively studied by measurement tests and modeling [2,39,44], which indicate that the model of radiated noise can be the same with large ships. Since an AUV’s prop does not rotate sufficiently fast to cause cavitation, it is hard to be detected with broadband noise in the high-frequency range. As is known, the low frequency harmonics are quite important for remote passive detection, and the experimental study in this work is set up with a very low frequency measurement by ocean bottom seismometer (samapling frequency $f_{s}=$200 Hz).

## 3. Adaptive Intrawell Matched Stochastic Resonance

### 3.1. Generalized Matched Stochastic Resonance with Duffing Oscillator

The bistable SR phenomenon can be described as a particle driven by periodic force and random force in a quartic double-well system, where the periodic motion can be heightened with the assistance of moderate noise. This can be governed by a two-dimensional Duffing oscillator as below [24],

(3) $$\frac{d^{2}x}{dt^{2}}+\gamma \frac{dx}{dt}=-\frac{dV(x)}{dx}+s\left(t\right)+\xi \left(t\right)$$

where γ is the damping factor, s(t) represents the input periodic signal, and n(t) stands for the noise item with noise intensity *D*. In particular, Equation (3) describes the movement of a particle in a quartic double well potential V(x),

(4) $$V\left(x\right)=-\frac{a}{2}x^{2}+\frac{b}{4}x^{4},a,b>0$$

in which *a* and *b* are barrier parameters of bistable SR system, and the potential barrier can be calculated by $\Delta V=a^{2}/\left(4b\right)$. As the analytic form of the SR output cannot be easily obtained, it is generally numerically calculated through the discrete fourth-order Runge-Kutta method [23]. Mathematically, let $\frac{dx}{dt}=y$, then Equation (3) can be separated into two first-order differential equations as,

(5) $$\begin{array}{c} \frac{dx}{dt}=y\\ \frac{dy}{dt}=ax-bx^{3}-\gamma y+s\left(t\right)+\xi \left(t\right) \end{array}$$

and then the output discrete time series x[n] corresponding to Equation (3) can be calculated by,

(6) $$\begin{array}{c} \left\{\begin{array}{c} y_{1}=y\left[n\right]\\ x_{1}=-V^{\prime }\left(x\left[n\right]\right)-\gamma y_{1}+s\left[n\right]+\xi \left[n\right]\\ y_{2}=y\left[n\right]+x_{1}h/2\\ x_{2}=-V^{\prime }\left(x\left[n\right]+y_{1}h/2\right)-\gamma y_{2}+s\left[n\right]+\xi \left[n\right]\\ y_{3}=y\left[n\right]+x_{2}h/2\\ x_{3}=-V^{\prime }\left(x\left[n\right]+y_{2}h/2\right)-\gamma y_{3}+s\left[n+1\right]+\xi \left[n+1\right]\\ y_{4}=y\left[n\right]+x_{3}h\\ x_{4}=-V^{\prime }\left(x\left[n\right]+y_{3}h\right)-\gamma y_{4}+s\left[n+1\right]+\xi \left[n+1\right]\\ x\left[n+1\right]=x\left[n\right]+\left(y_{1}+2y_{2}+2y_{3}+y_{4}\right)h/6\\ y\left[n+1\right]=y\left[n\right]+\left(x_{1}+2x_{2}+2x_{3}+x_{4}\right)h/6 \end{array}\right\} \end{array}$$

where *h* is the calculation step of the Runge-Kutta method.

From the perspective of parameter tuning, it can be considered as a special nonlinear filter, and the main issue is to achieve an “matched filter” output [24]. In general, a bistable system response is optimized by maximizing the signal-to-noise ratio improvement (SNRI) on system potential parameters *a* and *b*, time scaling parameter *h*, and damping factor γ. In this way, the nonlinear matched SR filter can be generally modeled with a generalized time-scale matching constraint as,

(7) $$\begin{array}{c} \begin{array}{cc} \max_{a,b,\gamma ,h} & \mathrm{SNRI}\\ \;s.t. & r_{K}=\frac{\Omega }{\pi }\\ \; & \gamma \in (0,1],\;\;h\in (0,1] \end{array} \end{array}$$

in which $r_{K}=\frac{a}{\sqrt{2}\pi \gamma }\exp\left(-\frac{a^{2}}{4bD}\right)$ is the famous Kramers rate, $\Omega =2\pi f_{0}$ represents the angular frequency of periodic forcing. The constraint of $r_{K}=\Omega /\pi$ is the time-scale matching condition that represent the statistical synchronization of interwell transition [14]. It is necessary to point out that this generalized condition is not sufficient as other optimized factors are required to judge the occurance of SR.

For sinusoidal signals with additive noise, the SNRI corresponding to Equation (3) could have a maximum at $D_{SR}=\Delta V$, and can be equivalent to $\hat{D}=a_{opt}/4b_{opt}$ in according to [24]. And as a consequence, the mathematic model of SR based nonlinear matched filter can be rewritten as,   

(8) $$\begin{array}{c} \begin{array}{cc} \left(\gamma_{opt}^{*},h_{opt}^{*}\right) & =\underset{\gamma ,h}{\mathrm{argmax}}\;\mathrm{SNRI}\\ s.t. & a_{opt}=2\sqrt{2}\pi f_{0}\gamma e\\ \; & b_{opt}=a_{opt}^{4}/4\hat{D}\\ \; & \gamma \in \left(0,16\sqrt{2}\pi f_{0}e\right],h\in \left(0,1\right] \end{array} \end{array}$$

where e is the base of natural logarithms, and the optimal potential parameters $a_{opt}$ and $b_{opt}$ can be obtained with matched relationship that related to the frequency of periodic signal $f_{0}$, damping factor γ and the noise intensity estimator $\hat{D}$.

To adaptively adjust these system parameters, the measurement index of input-output SNR improvement (SNRI) is extensively utilized and can be estimated by measuring the power spectral of a time series. This can be further limited with a bandwidth ΔB for the local SNR ( ${\mathrm{SNR}}_{local}$) calculation as it is generally connected to the performance of signal detection [45]. Besides, evaluation indexes such as power spectrum kurtosis (PSK), peak SNR (PSNR), spectral correlation coefficient (SC), structural similarity index (SSI), etc. can be well adopted and fused [20].

### 3.2. Framework of Intrawell Matched Stochastic Resonance with Potential Constraint

The “classical” description of the SR phenomenon in a bistable system is that a particle can jump across the potential barrier back and forth in the assistance of proper noise. This is called interwell jumping behavior as the random-switching frequency $r_{K}$ (Kramers rate) is made to agree closely with the periodic forcing angular frequency [14,35]. From the view of parameter tuning, the adjustable potential parameters can be equivalent to tuning the potential depth ΔV and well location (stable focus) $\pm x_{m}$ [28], where Equation (4) can be transformed into the function of well location and potential depth parameter as,

(9) $$V\left(x\right)=-\Delta V\left[2{\left(\frac{x}{x_{m}}\right)}^{2}-{\left(\frac{x}{x_{m}}\right)}^{4}\right]$$

**Figure 1:**
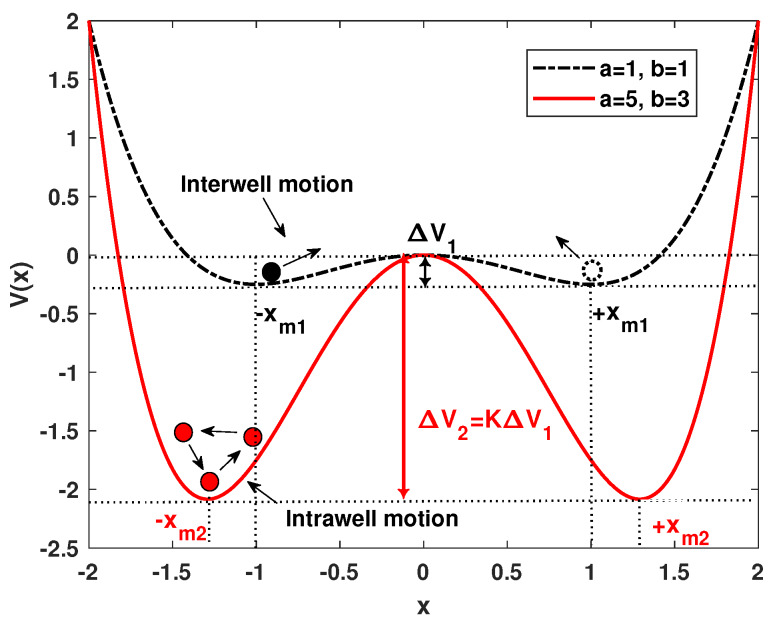
A demonstration of bistable stochastic resonance with interwell and intrawell motion.

In this way, changing the potential parameters *a* and *b* is essential to adjust the potential depth ΔV and the separation $2x_{m}$. A demonstration of particles in a bistable model with different potential parameters is given in Figure 1. It can be seen that a large potential depth can lead to longer system response time for transition, and even intrawell response [35,36].

As is known, the time-scale matching constraint is directly connected to the system response time. The nonlinear filtering effect of matched stochastic resonance can be essentially regarded as a proper control of the particle’s response. From this view, it is easy to know that the more the degrees of freedom for parameter tuning, the better the output response. Such results can be found in plenty of SR-related publications with multi-parameter optimization. The classical Duffing equation model with bistable potential utilizes parameter *a*, *b*, and γ are used to characterize the system response. It is intuitive to add new degrees of freedom for better characterizing and controlling of the particle’s response. Hence, a potential constraint is further adopted here to ease the insufficient time-scale matching constraint [37]. By this means, the mathematical framework of matched stochastic resonance can be further generalized by intuitively adding a constraint of barrier factor *K* as below,

(10) $$\begin{array}{c} \begin{array}{cc} \left(\gamma_{opt}^{*},h_{opt}^{*},K_{opt}^{*}\right) & =\underset{\gamma ,h,K}{\mathrm{argmax}}\;\mathrm{SNRI}\\ \;\;\;\;\;\;s.t. & a_{opt}=2\sqrt{2}\pi f_{0}\gamma e\\ \; & b_{opt}=a_{opt}^{4}/4K\hat{D}\\ \; & \gamma \in \left(0,16\sqrt{2}\pi f_{0}e\right],h\in \left(0,1\right],K\in \left(1,K_{max}\right] \end{array} \end{array}$$

in which *K* should be a postive real number to adjust the particle motion, and can be optimized from a proper searching interval.

The merits of this potential constraint model can be summarized as follows: (1) it can ease the insufficient time-scale matching constraint so as to weaken the uncertain effect on potential parameter tuning; (2) the inaccurate noise intensity estimation can be eased as well; (3) it can release the limitation on a system response which allows us a higher input frequency in breaking through the large sampling rate limitation. All in all, the potential constraint model is anticipated to be superior in nonlinear filtering performance, especially under a low sampled circumstance that is highly concerned for engineering applications.

### 3.3. Adaptive Strategy for Optimized Implementation

As mentioned in Equation (10), it is a multi-parameter optimization problem, where the constraint of the matched potential relationship is a nonlinearity that can properly be solved by intelligent optimization algorithms such as genetic algorithm (GA), particle swarm optimization (PSO), ant colony optimization (ACO), grey wolf optimization (GWO), et al. The main problem is to determine the proper searching interval of each parameter.

To address this problem, it can be known from [24,37] that in matching high frequency, signals require extremely large system parameters. As for practical engineering fields, the frequency of received signals generally varies from tens to thousands of Hertz, and hence, the order of magnitude for a matched potential parameter should be extremely large, especially for parameter *b* as there is a relationship with $b\propto a^{4}$. This problem is the same to the barrier factor *K* as there is an experimental guidance that $K_{opt}^{*}$ approximate to $f_{0}^{4}$ [37]. This refers to a large range of parameter searching interval to limit the computational efficiency and parameterized realization. Such a problem will limit the utilization in a real-time online embedded system in confronting data overflow problem (such as commonly utilized 32 bits digital signal processor (DSP)). From this view, the frequency shift to low frequency should be a kind of efficient solution for this problem, while lack of flexibility in dealing with unknown frequency signal or multi-frequency signal. In the consideration of the degree of freedom, the potential constraint of a matched relationship can be relaxed by the new freedom of the barrier factor *K*. As a consequence, the matched potential relationship can be eased. From the aforementioned analysis, for this problem, one can use the biquadrate relationship between the potential parameter *a* and *b*. In this regard, we can assume a particular case here by easing the potential parameter $a_{opt}=1$. As a result, the computation complexity is greatly deduced, and the extremely large parameter limitation is relaxed simultaneously. The simplified model can be expressed as,

(11) $$\begin{array}{c} \begin{array}{cc} \left(h_{opt}^{*},K_{opt}^{*}\right) & =\underset{\gamma ,h,K}{\mathrm{argmax}}\;\mathrm{SNRI}\\ \;\;\;\;\;\;s.t. & a_{opt}=1\\ \; & b_{opt}=1/\left(4K\hat{D}\right)\\ \; & \gamma_{opt}={\left(2\sqrt{2}\pi f_{0}e\right)}^{-1}\\ \; & h\in \left(0,1\right],\;\;K\in \left(0,K_{max}\right] \end{array} \end{array}$$

where the $K_{max}$ can be further limitted, and the optimal damping factor $\gamma_{opt}$ is underdamped as well. Here, we need to point out that in this simplification process, the insufficient time-scale matching constraint result in an insufficient relation of $\gamma_{opt}={\left(2\sqrt{2}\pi f_{0}e\right)}^{-1}$. Therefore, this constraint should be eased by a generalized underdamped condition to keep the degrees of freedom. Hence, the optimization model can be further expressed as

(12) $$\begin{array}{c} \begin{array}{cc} \left(\gamma_{opt}^{*},h_{opt}^{*},K_{opt}^{*}\right) & =\underset{\gamma ,h,K}{\mathrm{argmax}}\;\mathrm{SNRI}\\ \;\;\;\;\;\;s.t. & a_{opt}=1\\ \; & b_{opt}=1/\left(4K\hat{D}\right)\\ \; & \gamma \in \left(0,1\right],\;\;h\in \left(0,1\right],\;\;K\in \left(0,K_{max}\right] \end{array} \end{array}$$

### 3.4. Implementation of Adaptive Intrawell Matched Stochastic Resonance

In the last subsection, we have the adaptive strategy as an optimization problem with nonlinear constraints. Here, a signal processing strategy is proposed by jointly optimizing the parameters with a classical genetic algorithm (GA) method for the sake of global optimality. Consequently, the detailed implementation steps are summarized as follows,

(1) Signal pretreament. Data normalization and prewhitening are executed on the actual received noisy signals.

(2) Searching range initialization. Initialize the optimization searching range of parameters γ∈[0,1], h∈[0.001,1] and K∈[0.001,200], respectively.

(3) Parameter optimization. Obtain the optimal parameters $\gamma_{opt}$, $h_{opt}$, and $K_{opt}$ according to the following objective function,

(13) $$\begin{array}{c} \begin{array}{cc} \left(\gamma_{opt}^{*},h_{opt}^{*},K_{opt}^{*}\right) & =\underset{\gamma ,h,K}{\mathrm{argmax}}\;\mathrm{SNRI} \end{array} \end{array}$$

where SNRI corresponds to the fitness criteria of GA method. Here, the input–output SNR improvement (SNRI) is constructed by the superposition of global SNRI (${\mathrm{SNRI}}_{global}$) and local SNRI (${\mathrm{SNRI}}_{local}$) to better characterize the filtering performance both in the view of global and local conditions. The SNR calculation for the input and output time series can be defined as,

(14) $$\mathrm{SNR}=10\log_{10}\frac{P_{s}-P_{n}}{P_{n}}$$

in which $P_{s}=\sum_{i=f-\Delta B_{s}/2}^{f+\Delta B_{s}/2}S_{i}$ represent the total power around the characteristic frequency $f_{0}$ within a small bandwidth of $\Delta B_{s}$, and $P_{n}=\frac{1}{\Delta B}(\sum_{i=f-\Delta B/2}^{f+\Delta B/2}S_{i}-P_{s})$ represent the average power of background noise within ΔB bandwidth around the characteristic frequency $f_{0}$. For the global SNR and the local SNR calculation, ΔB is setted by the full bandwidth and $f_{s}/100$, respectively.

For a multi-harmonic signal circumstance, a modified measurement index of average SNRI is utlized, which can be defined as,

(15) $$\mathrm{SNRI}=\frac{1}{M}\sum_{h=1}^{M}\mathrm{SNRI}(h)$$

where *M* is the harmonic number of the received signal. The parameter optimization algorithm is summarized in Algorithm 1.

(4) Signal post-treatment. Output optimal waveform and the coresponding optimal fittest value.
**Algorithm 1Parameter Optimization Algorithm.**
 **Parameter Initialization:** $C_{r}=\left[\gamma_{start}=0,h_{start}=0.001,K_{start}=0.001;\gamma_{end}=1,h_{end}=1,K_{end}=200\right]$: the searching intervals; $N_{c}=3$: number of chromosome; $N_{p}=100$: Number of individuals in the population; χ=0.95: The fraction to be replaced by crossover in each iteration; μ=0.01: The mutation rate; M=10: The maximal iteration times; $\lambda_{stop}=0$: The threshold of stop condition. **Initialize generation** 0: *k*:=0; $P_{k}$:=a population of $N_{p}$ randomly-generated individuals;
 **Evaluate**
$P_{k}$:
 Compute fitness criteria SNRI for each $i\in P_{k}$;{ 1: Compute the corresponded MSR output by fourth order Runge–Kutta (RK4) method according to    Equation (6) and obtain x[n](n=1,2,…,N), where N is the length of the time series; 2: Compute the SNRI according to Equation (14) and Equation (15);} **Create generation** k+1: **do**{ 1: **Copy**: Select (1−χ)∗n members of $P_{k}$ and insert into $P_{k+1}$; 2: **Crossover**: Select χ∗n members, pair them up to produce offspring and insert the offspring into $P_{k+1}$; 3: **Mutate**: Select μ∗n members of $P_{k+1}$, and invert a randomly selected bit; 4: **Evaluate $P_{k+1}$**; 5: **if**
$P_{k+1}-P_{k}\leq \lambda_{stop}$
**then** break; 6: **else** 7:   **Increment:** k:=k+1; 8: **end if**} **while**
k⩽M; **return** the optimal fittest individual from $P_{M}$;

## 4. Filtering Performance Analysis and Evaluation

SR can be regarded as a specific kind of nonlinear filter, while generally requires a high sampling rate condition. As is noticed in the Section 3.2, the proposed method can release the limitation on system response so as to allow us a higher input frequency in breaking through the large sampling rate limitation. Hence, here we intuitively exhibit the filtering performance referred to the discrete line signature and harmonic related line signature under a low sampling rate condition.

### 4.1. Discrete Line Signature Signal Analysis

Without loss of generality of detection, we consider two circumstances corresponding to $H_{1}$ and $H_{0}$ hypotheses, respectively. The sampling frequency is set to $f_{s}=100$ Hz, and the data length N=2048.

**Figure 2:**
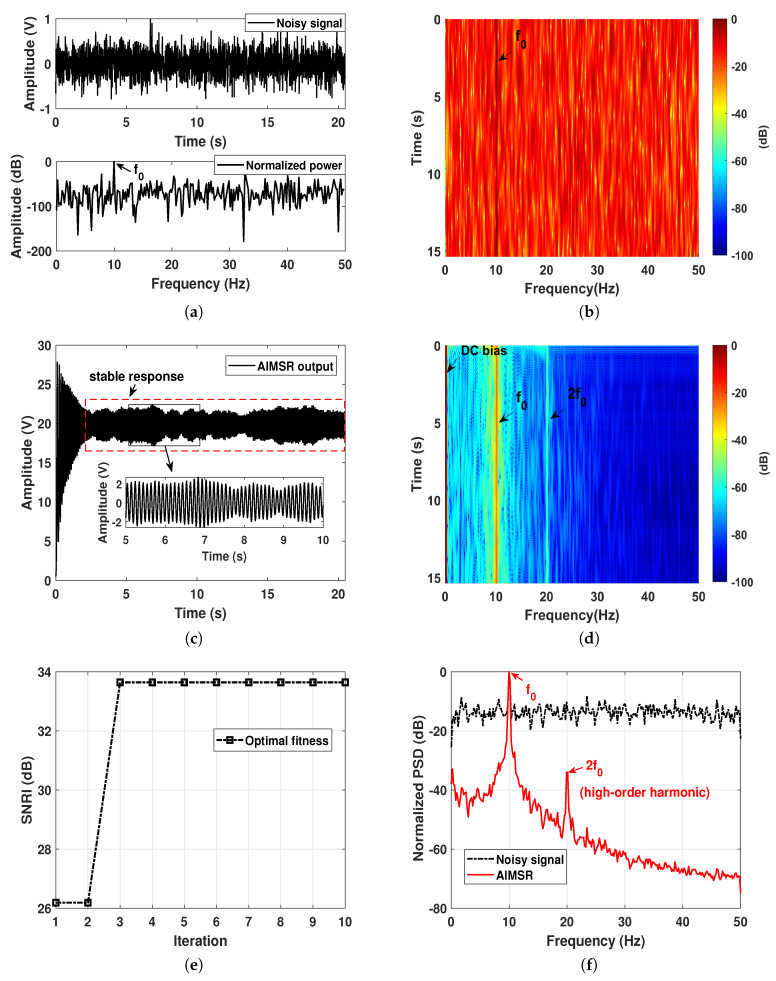
A simulation of discrete line signature signal on $H_{1}$ assumption: (**a**) the input waveform and its normalized power spectrum; (**b**) lofargram obtained with the input; (**c**) the adaptive intrawell matched stochastic resonance (AIMSR) output waveform ($\gamma_{opt}=0.0322$, $K_{opt}=79.05$, $h_{opt}=0.4413$); (**d**) lofargram obtained with the AIMSR output; (**e**) the optimal fitness at each iteration with genetic algorithm (GA) method; (**f**) filtering performance comparison with normalized power spectrum density (PSD, Welch method).

For $H_{1}$ hypothesis circumstance, the input is composed of a narrowband component with the frequency $f_{0}=10$ Hz, and the ambient noise that simulated by the Gaussian noise. The input SNR is set as −15 dB, and the normalized waveform and power spectrum are shown in Figure 2a. First, we employed the lofargram to qualitatively evaluate the performance. A 2.56 s short-time Fourier transformation (STFT) with the overlapping length of 0.1 s was used to generate the lofargrams. The lofargrams of the original input is illustrated in Figure 2b, where we can see that the narrowband component is not easy to be identified. By utilizing the proposed AIMSR method, the optimal output waveform can be seen in Figure 2c, where it reflects an intrawell response with large amplitude. After a response delay, it is stable and periodic at 10 Hz. The lofargram of the AIMSR output is demonstrated in Figure 2d. It clearly identifies the narrowband component $f_{0}$. It is worth noting that there is a second-order harmonic with periodic driving. This can be attribiuted to the resonance phenomenon and anticipate benefiting harmonic signature extraction. A comparison of normalized power spectrum density (PSD) of input and output (Note a highpass filter is adopted with passband frequency 1 Hz to deal with the DC bias, and this is the same in the rest of the paper) with the Welch method is given in Figure 2f, where we can see the superior filtering performance. The frequency response of the AIMSR background noise has the form of Lorentzian distribution by concentrating most of the noise energy into the low-frequency region. This refers to the special property of nonlinear SR phenomenon. The optimal SNRI obtained at each iteration is shown in Figure 2e. It can be seen after three iterations that an optimal result is obtained, which reflects a convergence of the proposed algorithm.

**Figure 3:**
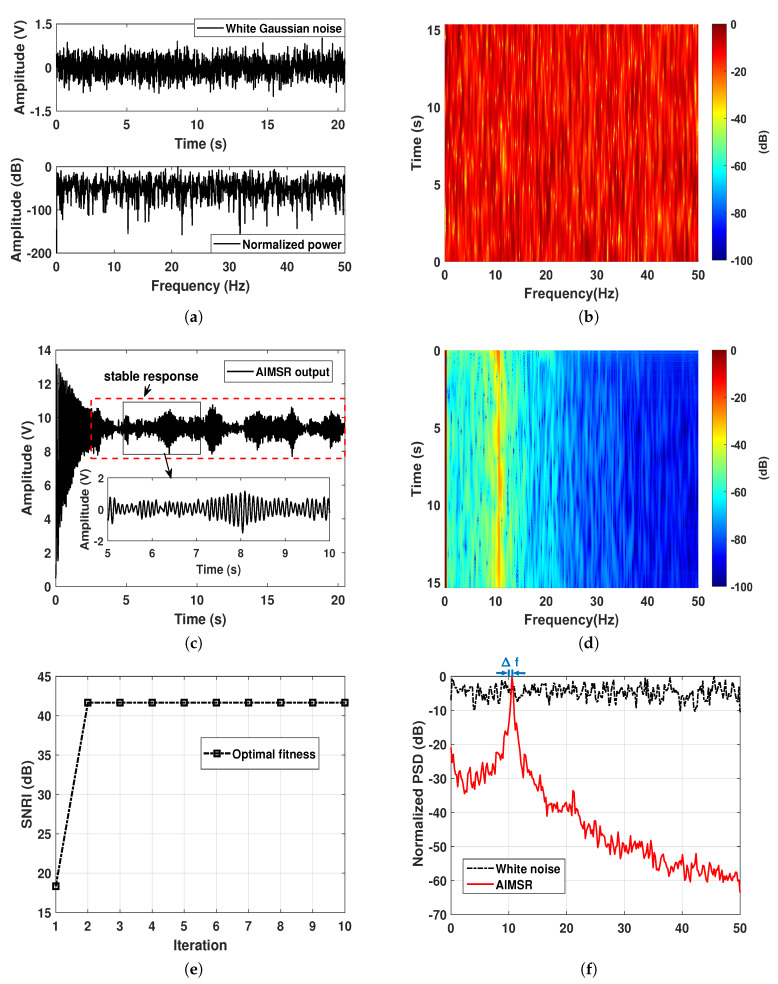
A simulation of discrete line signature signal on $H_{0}$ assumption: (**a**) the input waveform and its normalized power spectrum; (**b**) lofargram obtained with the input; (**c**) the AIMSR output waveform ($\gamma_{opt}=0.2875$, $K_{opt}=109.4507$, $h_{opt}=0.4465$); (**d**) lofargram obtained with the AIMSR output; (**e**) the optimal fitness at each iteration with GA; (**f**) filtering performance comparison with normalized PSD (Welch method).

For $H_{0}$ hypothesis circumstance, the input is simulated by the pure white Gaussian noise. As is assumed by prior unknown, we conduct the optimization with $f_{0}=10$ Hz for parameters initialization as well. The corresponding results are demonstrated in Figure 3a–f. By utilizing the proposed AIMSR method, the optimal output waveform is shown in Figure 3c, where it reflects an intrawell response as well. After a response delay, the amplitude looks more fluctuant compared with $H_{1}$ hypothesis circumstance as illustrated in Figure 2c. The lofargram of the AIMSR output is demonstrated in Figure 3d, where there seems to be a narrowband component near 10 Hz. To further compare the normalized power spectrum density (PSD) of the input and output in Figure 3f, we can see a frequency bias with Δf that does not match the initialized frequency $f_{0}$. Such a circumstance does not have a high-order harmonic that is regarded as a non-SR occurrence. The optimal SNRI obtained at each iteration is shown in Figure 3e, where the optimal result is obtained after two iterations. The optimal fitness value is larger than $H_{1}$ hypothesis circumstance. This should be affected by the calculation result of input as the input SNR is extremely smaller.

In summary, it is clear to see the superior filtering performance of AIMSR with a limited sampling rate condition. The difference in response between the $H_{1}$ and $H_{0}$ hypotheses can be identified, which indicates the detection performance fairly well.

### 4.2. Harmonic Related Line Signature Signal Analysis

Harmonic vibration is a typical vibration generated by rotatory machinery. As is mentioned in Section 2, the harmonic-related signature is commonly referred to in a ship engine and propeller. The sampling frequency is set to be $f_{s}=100$ Hz, and the data length N=2048. We consider two circumstances corresponding to $H_{1}$ and $H_{0}$ hypotheses as well.

**Figure 4:**
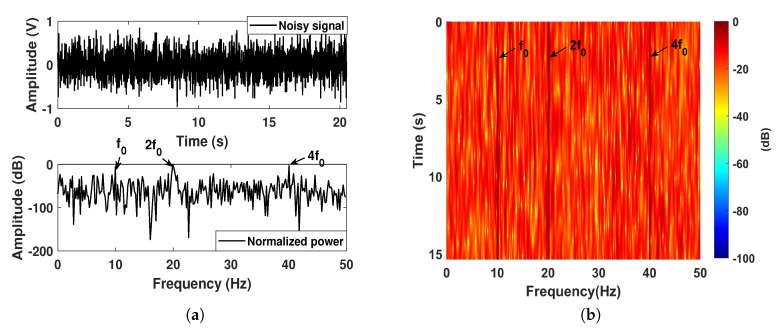

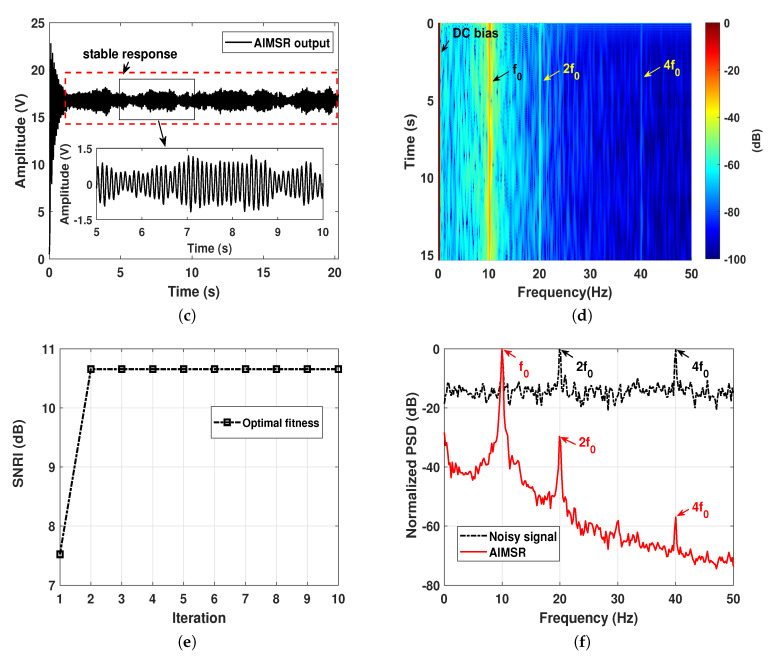
A simulation of harmonic-related line signature signals on $H_{1}$ assumption: (**a**) the input waveform and its normalized power spectrum; (**b**) lofargram obtained with the input; (**c**) the AIMSR output waveform ($\gamma_{opt}=0.1132$, $K_{opt}=70.4507$, $h_{opt}=0.4429$); (**d**) lofargram obtained with the AIMSR output; (**e**) the optimal fitness at each iteration with GA; (**f**) filtering performance comparison with normalized PSD (Welch method).

For $H_{1}$ hypothesis circumstance, the tested harmonic signal is a combination of a fundamental sinusoid and its two high-order harmonics, whose frequencies are all harmonically related to two times and four times the integer multiples of the fundamental frequency $f_{0}$. These three sinusoidal signals have the same amplitude of 0.1, and the same Gaussian noise background. The input SNR is set as −15 dB. The lofargram of the original input is illustrated in Figure 4b, where we can see the three harmonic-related narrowband components. By utilizing the proposed AIMSR method, the optimal output waveform still responds well with a response delay as shown in Figure 4c. In comparing with the single periodic driving, the amplitude of stable response is smaller. The corresponding lofargram of the AIMSR output is demonstrated in Figure 4d, where the three harmonic-related narrowband components can be identified. To further evaluate the filtering performance with normalized power spectrum density (PSD) of input and output in Figure 4f, we see the great local denoising performance with the fundamental frequency $f_{0}$, which is gradually lost with the 4th order harmonic ($4f_{0}$). The optimal SNRI obtained after two iterations as shown in Figure 4e, where we can find the optimal value is so small compared with the discrete line signature signal circumstance. There is a negative improvement of global SNRI for the high order harmonics. In this way, the local SNRI is recommended to be the evaluation index of high-order harmonics.

**Figure 5:**
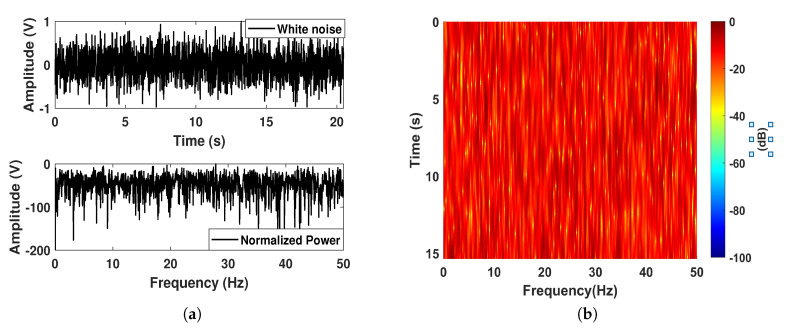

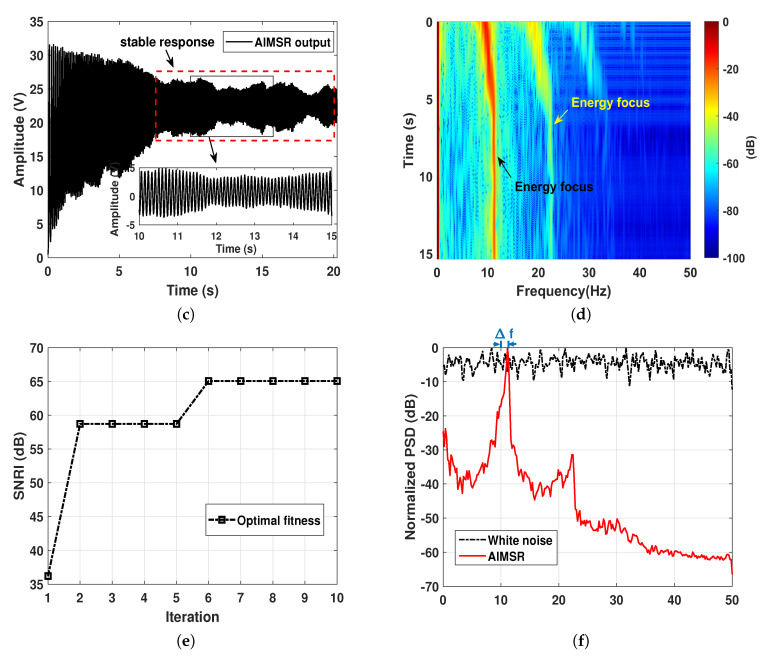
A simulation of a harmonic-related line signature signal on $H_{1}$ assumption: (**a**) the input waveform and its normalized power spectrum; (**b**) lofargram obtained with the input; (**c**) the AIMSR output waveform ($\gamma_{opt}=0.0462$, $K_{opt}=159.2903$, $h_{opt}=0.5763$); (**d**) lofargram obtained with the AIMSR output; (**e**) the optimal fitness at each iteration with GA; (**f**) filtering performance comparison with normalized PSD (Welch method).

For the $H_{0}$ hypothesis, the input is simulated by the pure white Gaussian noise. As is assumed, we conducted the optimization with $f_{0}=10$ Hz for parameter initialization as well. The corresponding results are demonstrated in Figure 5a–f. By utilizing the proposed AIMSR method, the optimal output waveform is shown in Figure 3c. The output is an intrawell response with a larger response delay, and the amplitude of stable response reflects larger effects. The lofargram of the AIMSR output is demonstrated in Figure 5d, where we can see a broadband harmonic energy in the first 5 seconds, and then the energy is focused to a periodic mode with a frequency bias Δf that does not match the initialized frequency $f_{0}$ as shown in Figure 5f. This means no SR occurs as the noisy input can not be matched to the nonlinear system. The optimal fitness value is extremely large which is consistent with the $H_{0}$ hypothesis of the discrete line signature signal case. This indicates the detectability as well.

In summary, the proposed AIMSR method can be utilized to enhance the harmonic-related signature, especially for the fundamental frequency. Since the fundamental frequency estimation is a topic that spans many disciplines including passive sonars [39], speech recognition [46], biomedical signal processing [47], musical pitch estimation [48], and etc., this method is anticipated to be a potential new technique for the future. The difference in responses between the $H_{1}$ and $H_{0}$ hypotheses can be identified with proper measurement index to establish a detector. The computation is efficient and generally converges to an optimum within five iterations, which can be realized in the embedded system. This work will be a topic for further study in the future.

## 5. Application Verification and Discussion

### 5.1. Verfication on AUV’s Low Frequency Propeller Harmonic Tonals

**Figure 6:**
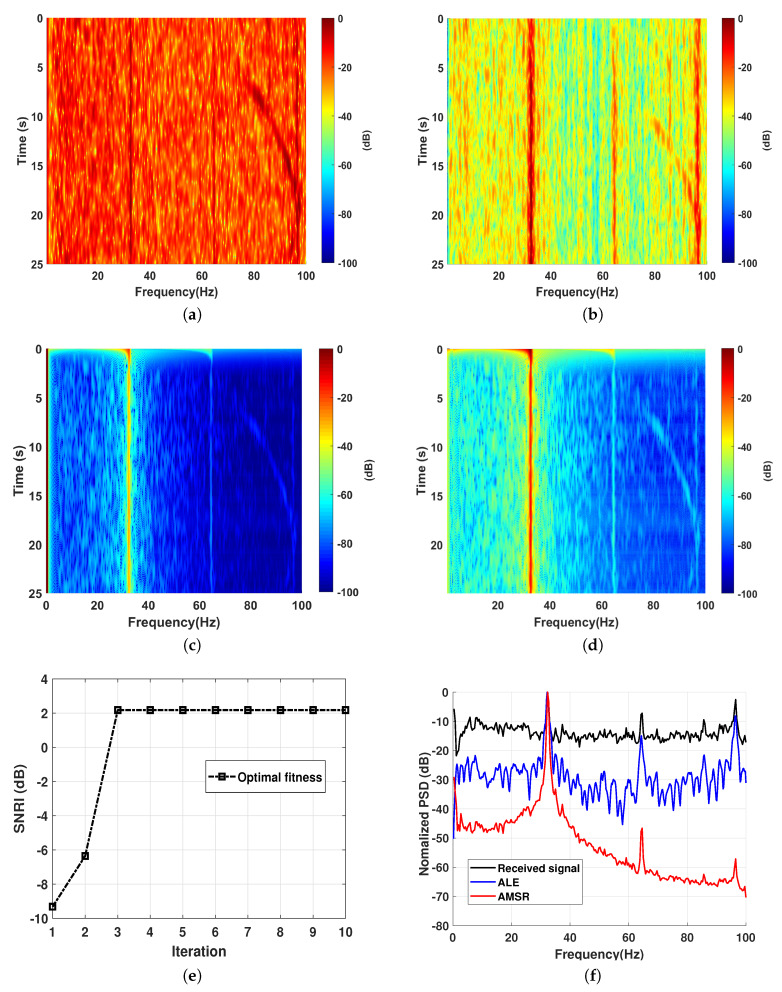
Filtering performance verification: (**a**) lofagram of the original received signal of an autonomous underwater vehicle (AUV); (**b**) lofagram of the adaptive line enhancer (ALE)output (LMS); (**c**) lofagram of the AMSR output ($\gamma_{opt}=0.1227$, $K_{opt}=85.6350$, $h_{opt}=0.4707$); (**d**) lofagram of the AMSR output with direct current (DC) offset filter; (**e**) the optimal fitness at each iteration with GA; (**f**) normalized power spectrum denisty (PSD) comparison.

To validate the feasibility and efficiency of the proposed method, an experiment was conducted with an AUV in the Zhanghe reservoir. The AUV moved away in a trajectory of straight line, and the radiated noise was measured by a low frequency seismometer with the sampling frequency $f_{s}=200$ Hz. Figure 6a shows the lofargram of the received signal, where we can see three harmonic lines. The conventional ALE that employs the least mean square (LMS) algorithm is conducted to enhance the line signatures as shown in Figure 6b, where the harmonic lines are more clearly identified. By utilizing the proposed AIMSR method, the optimal output is the intrawell as well. The corresponding lofagrams of the AIMSR output and the direct current (DC) offset filtered output are illustrated in Figure 6c,d, respectively. It can be seen that the background noise of the AIMSR output is much lower than that of the conventional ALE. A comparison of normalized power spectrum denisity (PSD) is shown in Figure 6f, where there is about 10 dB improvement for the fundamental frequency compared to that of the conventional ALE. For high order harmonics, the conventional ALE should be better. This indicates that a combination of the two methods gives a better output.

### 5.2. Discussion

(1) From the aforementioned numerical and practical analyses on AIMSR with potential constrainted bistable model, the SR is implemented under a low sampling rate condition. Essentially, it is to add a new degree of freedom to the bistable potential which can have an effect on the limitation of the system response. As we consider the system parameter tuning SR as a special nonlinear filter, it is a tenet that the more the tuning parameters (system complexity), the better output response. Similar results can also be found like multi-parameters tuning [49], improved potentials [30,50,51], coupled systems [31,32], etc. Hence, one of the contribution of this paper is to explain and lead the further explore of nonlinear filter. In addition, from the view of system nonlinearity, there seems to be a connection to deep neural networks for the system complexity. This might lead to an inspiration and possibility in training the deep SR network for applications, and gives us a guidance to better understand the innate character of deep learning from the view of nonlinearity as well.

(2) The evaluation index of SNRI in this paper generally requires a prior knowledge of signal frequency. However, for engineering applications, it is commonly unknown. As is known, the SR will always force the energy focus to a periodic mode. It is hard to distinguish whether the correctness of output response is simply achieved by frequency searching. Although the difference of response between the $H_{1}$ and $H_{0}$ hypotheses can be fairily well identified, the problem of proper selection of the measurement index in the sense of detection is still a problem for further studies.

(3) The superior filtering performance is validated in this paper, which means the advantage of AIMSR is a potential. However, its intrinsic Lorentzian property leads to a preference in enhancing the low-frequency signals, and as a result, to a loss of the performance for higher frequencies. As is mentioned in the last subsection, a conventional ALE should be better in dealing with the high-order harmonics. Proper combination on these two methods is needed for better outputs. This work will probably be studied in the future.

(4) The proposed AIMSR method is of low computational complexity which enables implementation in an embedded system. This is an interesting and highly important engineering problem. However, related work can rarely be found. To further improve the applicablity of the SR in the engineering fields, we think this work is necessary for future studies.

## 6. Conclusions

In this paper, we proposed an adaptive intrawell matched stochastic resonance (AIMSR) method to break through the limitation of the conventional ALE by a nonlinear filtering effect. The problem of parameterized implementation of SR under a low sampling rate condition is firstly addressed by implementing a framework of intrawell matched stochastic resonance with potential constraint. To further promote its practicality in an embedded system, the large parameter limitation of matched relationship is eased to deduce the computation complexity. Simulation analyses are conducted with a discrete line signature and harmonic-related line signature that reflect the superior filtering performance as well as the computational efficiency fairy well. Besides, two hypotheses corresponding to $H_{1}$ (periodic signal with noise) and $H_{0}$ (pure noise) circumstances are further considered to reveal the feasibility of detection. Application verification was experimentally conducted in a reservoir with an AUV to validate the proposed AIMSR method compared with the conventional ALE method. Additional intensive discussions have been made to give an insight into the principal results and inspire future investigations. In the end, we anticipate that the proposed method can be a potential new technique for passive sonar detection in the future.

## Figures and Tables

**Table 1:** Character frequencies of ship engine and propeller.

Engine Rates	Propeller Rates
Cylinder Firing Rate $f_{CF}=f_{CR}/2$	Shaft Rotation Rate $f_{SR}=f_{CR}/\lambda_{g}$ $\lambda_{g}$: Gear Ratio
Crankshaft Rotation Rate $f_{CR}=RPM/60$ RPM: Engine Speed	Blade Rotation Rate $f_{BR}=N_{b}f_{SR}$ $N_{b}$: Number of Blades
Engine Firing Rate $f_{EF}=Ncf_{CF}$ $N_{c}$: Number of Cylinders	
